# Advanced software concepts for employing microcomputers in the laboratory

**DOI:** 10.1155/S1463924679000407

**Published:** 1979

**Authors:** Scott B. Tilden, M. Bonner Denton

**Affiliations:** Department of Chemistry University of Arizona Tucson Arizona 85721 USA


### Advanced software concepts for employing microcomputers in the laboratory

**Scott B. Tilden and M.Bonner Denton,**
*Department of Chemistry, University of Arizona, Tucson, Arizona 85721, USA.*

### Introduction

While a proliferation of commercial chemical instrumentation is appearing today employing microprocessors for a variety of control and data reduction applications, the great potential of microprocessors has not been exploited extensively for individual custom applications. The primary reason for this phenomenon is altogether too clear – microprocessor software is either difficult to develop or inefficient in memory requirements and speed. This problem is even more important in situations requiring constant software modification. Initially, most instrument manufacturers utilized cross assemblers supported on large "number cruncher" computers to generate the required machine code binary program. More recently, the trend has been toward the use of a "developmental system" (at a cost comparable to a moderate minicomputer – the authors use the term "mini" in contrast to "micro" reluctantly because of the ever increasing overlap in computing capability) to write and debug assembly level porgrams which are subsequently converted to binary and incorporated into an instrument in the form of "read only memory" (ROM). While this approach

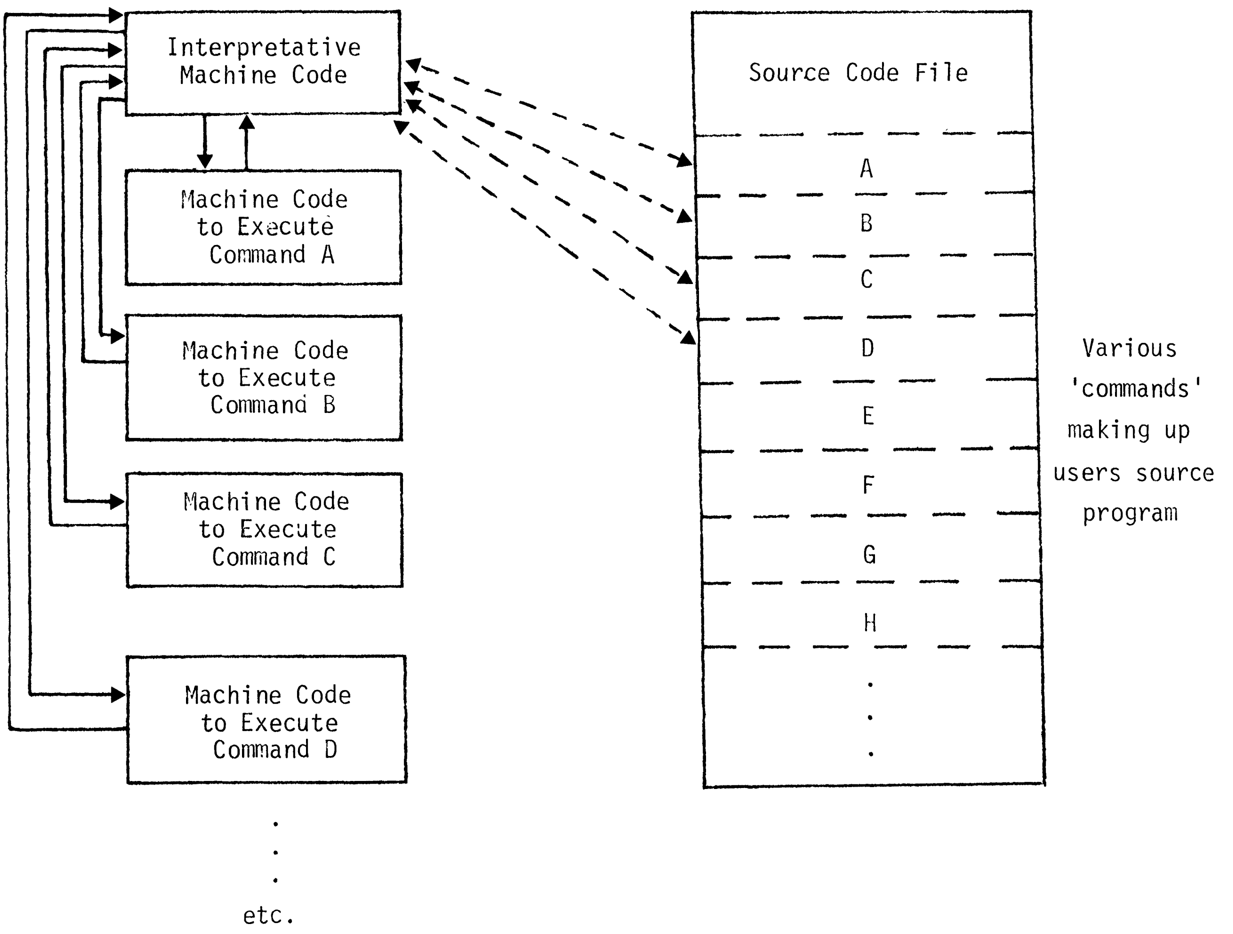

*Figure 1. The interpretative cycle of common types of languages such as* BASIC. *After examining each command in the source file, the interpreter searches for and branches to the corresponding block of machine code; thus, program execution always remains within the interpreter.*

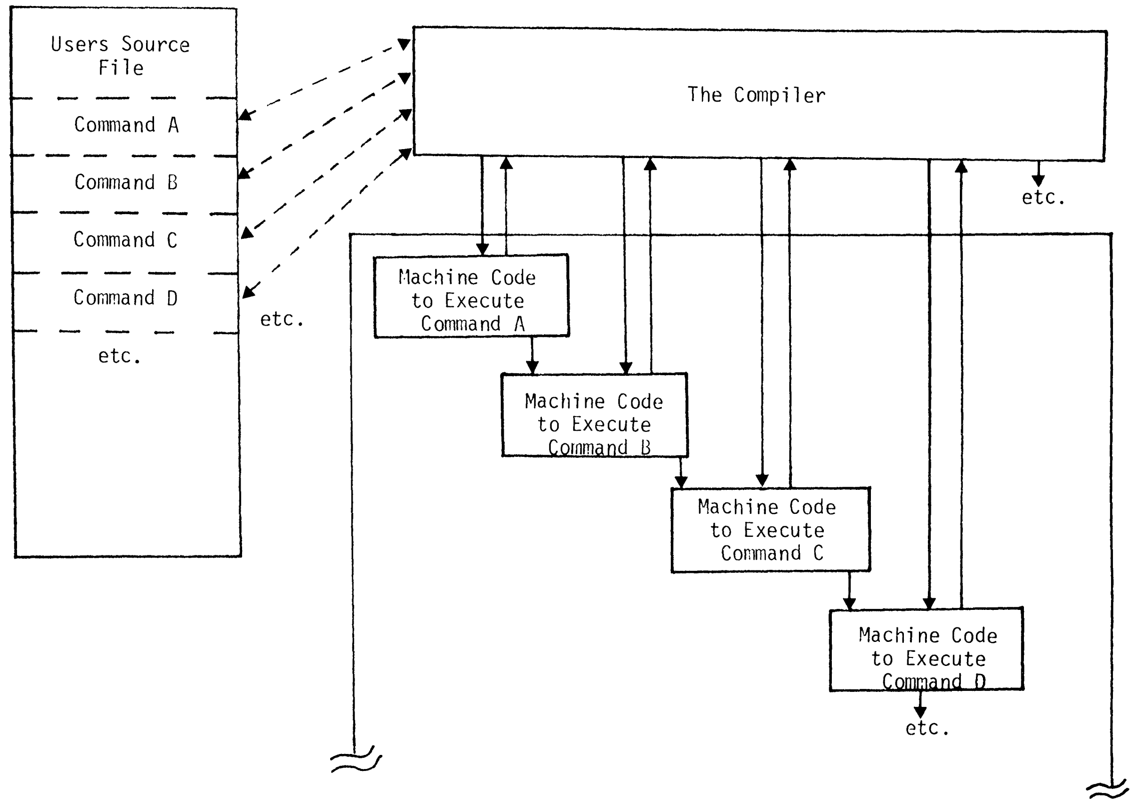

*Figure 2. Note that the compiler transforms each source "command" into executable machine code. This code will, subsequently, be loaded and executed independently of the compiler.*

has proven cost effective for high volume mass produced applications, it possesses serious limitations for system updates and custom applications. Additionally, the ability to program efficiently at the assembly level is a talent requiring a significant expenditure of time to develop.

During the past several years, a virtual deluge of sophisticated, flexible, high performance computer hardware has been introduced primarily aimed at a rapidly growing "hobbyist" market. Manufacturers quickly realilsed that to sell the public hardware, some form of reasonably high level software must be made available. A variety of BASIC interpreters, ranging from rather "dumb" to "quite intelligent" have since evolved. The more intelligent BASIC interpreters have several highly attractive attributes for "hobbyist" applications. The language is both easy to master and conversational. Error and caution messages are provided as aids during programming.

Why not apply the "hobbyist" technology toward the implementation of custom laboratory systems? Many investigators have and, no doubt, many more will take this approach. However, BASIC interpreters possess serious limitations in terms of system speed, flexibility and input/output (I/O) capabilities. In BASIC, each command must first be interpreted and then executed (see Figure 1). In many cases, the interpretation process takes much more time than the actual execution. This problem is compounded by the fact that commands interpreted in the past must be re-interpreted each time they are used causing iterative programs to be very slow. While speed is often not a serious limitation in playing computer games, laboratory application requiring high speed data acquisition and/or data manipulation are common. Additionally, the more intelligent BASICs make very inefficient use of memory often requiring a minimum of 12 or 16 K bytes (twelve or sixteen thousand eight bit words)'

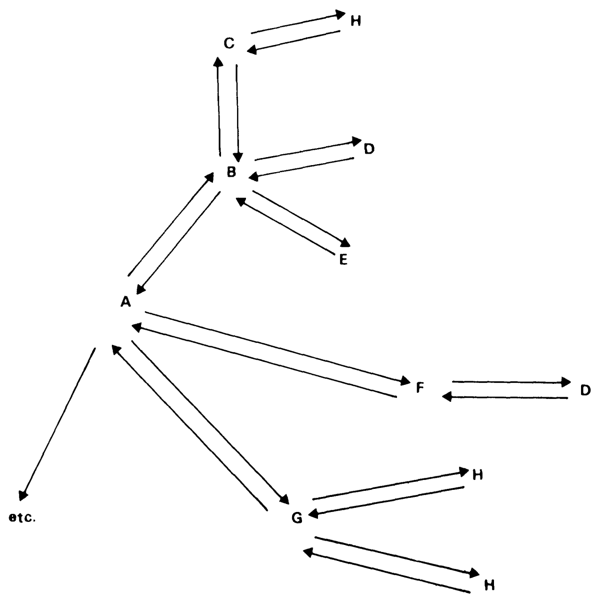

*Figure 3. The 'threaded' code approach used in CONVERS. In 'threaded' code programming modules for sub-routines are used repetitively in a variety of combinations to allow implementation of an infinite number of functions. Note that the flow of logic threads its way in a very non-linear fashion through these modules.*

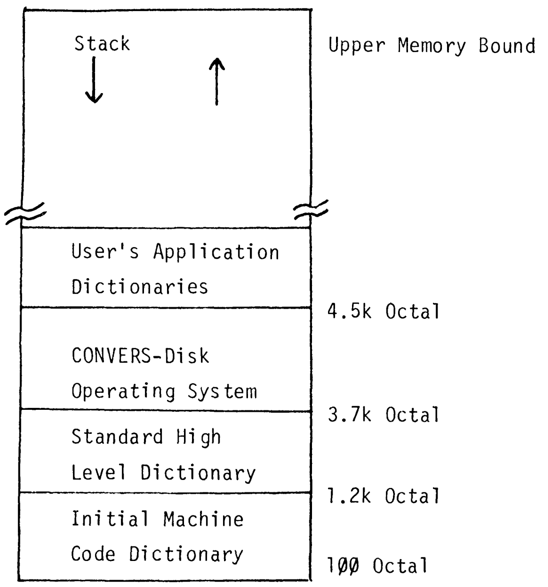

*Figure 4. Memory map of the CONVERS dictionary. The stack which is composed of data parameters etc. acts as a constantly expanding or contracting memory buffer which allows one sub-routine to communicate with another without either needing to know the other's location.*

In contrast to interpreters, high level compilers, such as FORTRAN, offer a much faster "run time" execution speed. This is accomplished through generation of the required machine code during a series of programming operations. Compilers using FORTRAN, which are designed to run on many minicomputers and some micros, often first transform user symbolic source code into assembly code. An assembler program, subsequently, transforms this into the required machine code. This ready-to-run machine code is often loaded along with a run time package which executes in the manner shown in Figure 2. While this approach greatly improves execution speed, the need for loading several different soft-ware routines increases the "hassle" associated with editing and debugging. Thus, this makes some form of mass memory, such as a disk or magnetic tape, almost mandatory. Additionally, I/O algorithms generally must be implemented in assembly level code!

One obvious question immediately arises – why not incorporate the most desirable characteristics of both interpreters and compilers into a single language? Additionally, due to the unique requirements found in many applications, why not allow the programmer additional flexibility by providing him with the ability to actually develop his own individual modifications and additions to the language itself? Other desirable features would include high memory efficiency, high level I/O programming, ease of understanding the language's "inter-working" and the ability to be transferred from one CPU to another with minimum effort.

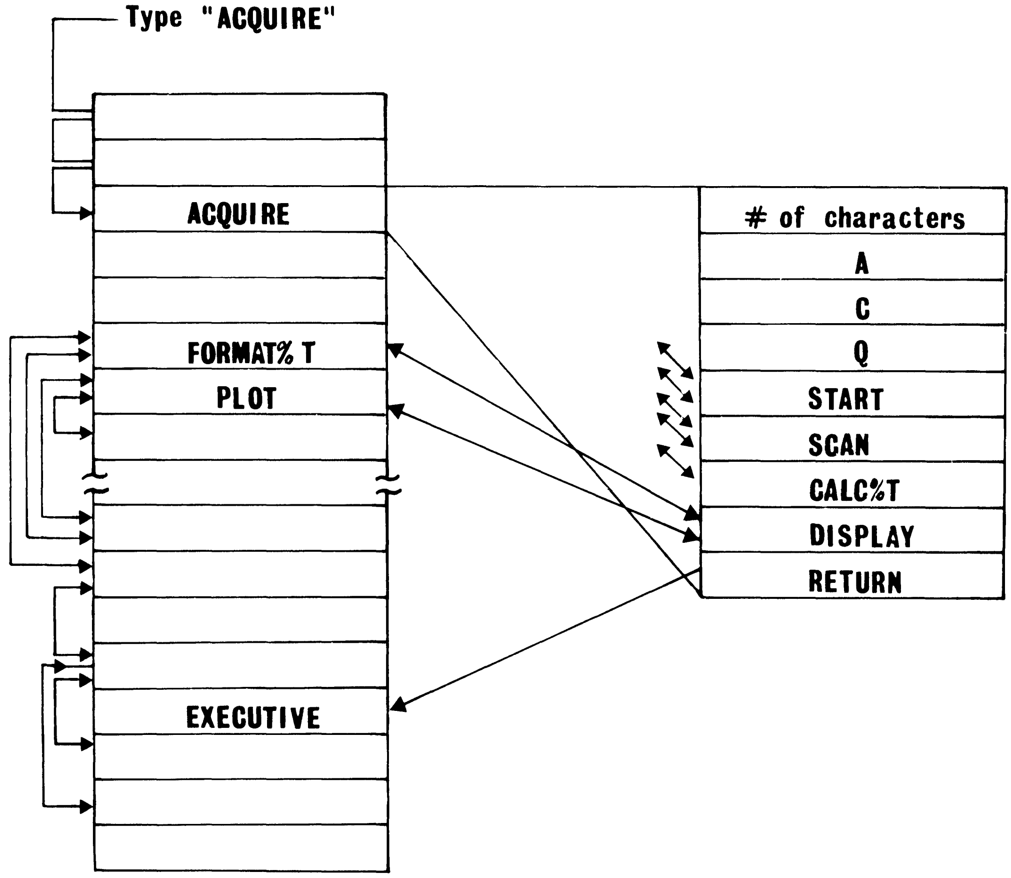

*Figure 5. If a previously defined program name (ACQUIRE) is entered when in EXECUTE mode, a dictionary search takes place locating the ACQUIRE entry. Once found, this entry contains all the required machine code and/or calls to addresses of other previously compiled machine code modules to completely execute the desired function.*

```
  2000  VARIABLE  START
  5000  VARIABLE  STOP
    20  VARIABLE  INCREMENT
     0  VARIABLE  LOCATION
:  A/D7  7  INDEVICE  ;
:  INITIALIZE  START  @  LOCATION  !  ;
:  STEP  LOCATION  @  INCREMENT  @  +  LOCATION  !  ;
:  DELAY  BEGIN-HERE  5  INDEVICE  10  AND  IF  A/D7  ELSE  BEGIN  THEN  ;
:  MOVE  LOCATION  @  5  OUTDEVICE  STEP  DELAY  ;
:  SCAN  INITIALIZE  BEGIN-HERE  MOVE  LOCATION  @  STOP  @  >  IF
END  ELSE  BEGIN  THEN  ;
  3000  START  !
  6500  START  !
    10  INCREMENT  !
  SCAN
```

*Figure 6. Ten lines of typical CONVERS code to scan wavelengths between two easily changed limits and acquire data. By reference to notes in the text it can be easily understood.*
*NOTE: a number or name pushes the number of address occupied by the name on the stack. The symbol @, pips the top number from the stack uses it as the address from which to obtain a number and pushes that number on the stack.*

### Development of CONVERS

During the past two years, a different approach to software has been taking place at the University of Arizona referred to as an "Interpretive Compiler" called CONVERS. This package, which is conceptually similar to the FORTH language currently being used in several minicomputer-astronomical applications [1], is able to provide many of the desirable features found in both interpreters and compilers by separating the compile and execute states (as a compiler does) while maintaining a resident user interactive and conversational executive which oversees system operation. The ability to realise such advanced software capabilities in a very modest amount of memory (less than 4 K bytes on an 8080 based micro) is the direct result of exploiting threaded code programming techniques (see Figure 3). The approach involves highly efficient use of simple macro-instructions to build more complex subroutines which are recombined with additional macroinstructions to form super subroutines. This process of combining previously defined modules to form ever increasingly sophisticated routines for performing the task at hand is the essence of threaded code programming. When initially loaded and running, CONVERS acts much like an interpreter, i.e. it is conversational, ready to either execute a previously programmed algorithm or accept a new one. However, in contrast to BASIC, when a new program is being entered under CONVERS, it is immediately transformed into binary machine code or to the binary starting addresses of other previously entered and compiled machine code programs. During this process, the operator is kept informed of the status of the program by a series of error and diagnostic messages. When the new program has been completed, it is entered in a program library or dictionary, which is constantly building up from low memory (see Figure 4). If the operator now wants to execute this program, he can request it from his terminal. A dictionary search will begin at the last entry and progress until the requested program is located. Once located, the requested program will run in its entirety without need for any additional dictionary searches. For example, let us assume an algorithm, called ACQUIRE, has been programmed to take data from some hypothetical experimental system. When ACQUIRE is requested from the terminal, a dictionary search is initiated. The program names ACQUIRE (see Figure 5), once located, contains the starting addresses of a series of previously defined modules which implement the various steps necessary to perform the desired experiment. For example, the module SCAN which might be intended to scan a monochromator's wavelength in some desired manner has been previously defined and tested. This ability to easily test each module separately and then efficiently combine a series of modules to perform a more complex function, test this function, and then employ it in a vastly more complex function, etc., i.e. testing each step as the threaded code is made increasingly complex, is a major factor contributing to the speed with which software can be developed using CONVERS. Use of a software stack also contributes toward improved memory efficiency and simplified programming.

The stack is an area of memory set aside to handle parameters, data numbers, etc. One of the primary advantages of the stack is that entries can leave temporary parameters on the stack without having to assign specific

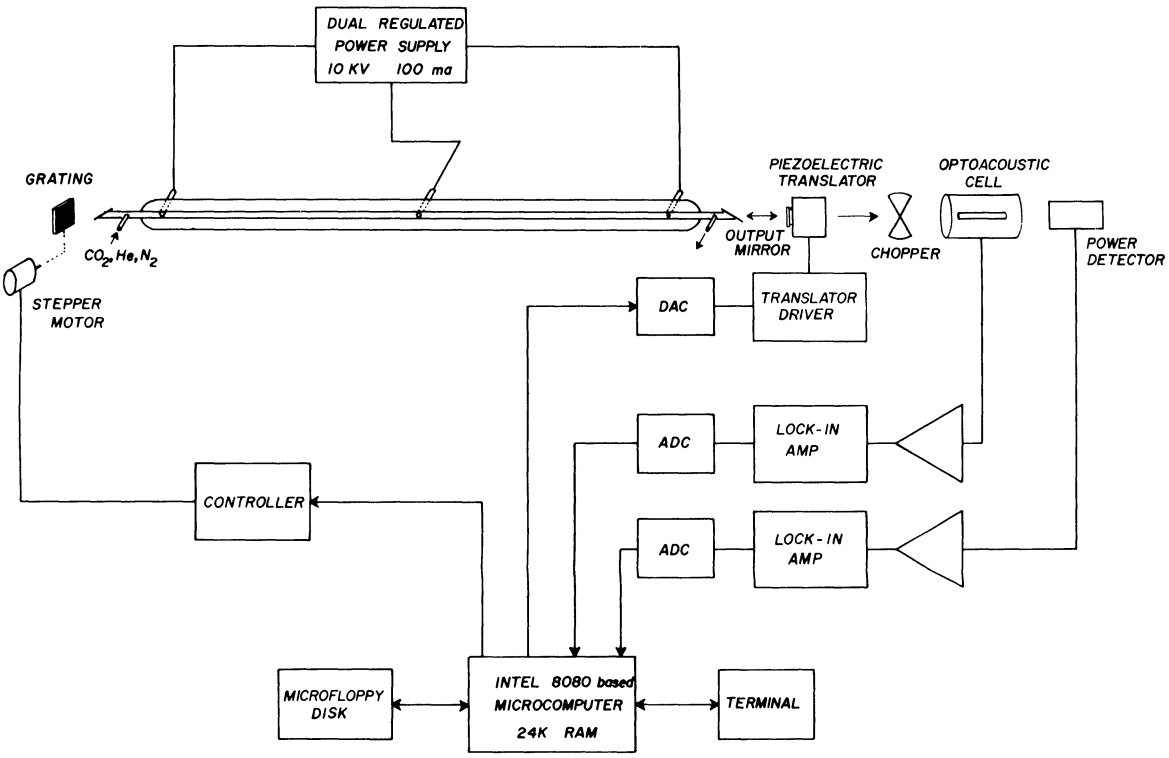

*Figure 7. The optoacoustic experiment in which a microcomputer is used to control laser wavelength and to monitor laser power and optoacoustic signal.*

memory locations to store them. This not only can save considerable memory, but also allows programs to be easily relocatable since one algorithm need only know that a previous routine left so many words of data, etc. on the stack. It need not know where the previous routine is nor even where the stack is located. A series of stack handling routines, which should appear quite familiar to many small calculator users, provide an array of capabilities, including the ability to PUSH a number on the stack, POP it off, duplicate it, SWAP the top two numbers, locate a number some distance into the stack, and copy it on top of the stack, etc. Additionally, a variety of logic functions familiar to the minicomputer user are provided including OR, AND, shift left, shift right, greater than, less than, etc.

Input/output (I/O) is normally accomplished using the stack in conjunction with the INDEVICE or OUTDEVICE commands. For example, to take data from a device located at I/O, port 7, the number seven is "pushed" onto the stack (7), goes to this I/O port, takes in a number and "pushes" the number on the stack. OUTDEVICE functions in a similar manner, requiring the number to be sent to the desired device to be "pushed" onto the stack followed by the device's I/O port address. Hence, to send the number 131 to device 11, the number 131 is pushed on the stack followed by 11 and then OUTDEVICE. This "pops" the top number (11) from the stack, uses it as the output port and then sends the number 131 to that location.

### Applications of CONVERS

To appreciate the ease with which real programs can be written, a few examples will be considered. A trivial program, called SOUND, which rings the terminal bell three times, might be written:

```
: SOUND BELL BELL BELL ;
```

The colon denotes changing from EXECUTE to COMPILE mode. After typing the name of the new routine, in this case to be called SOUND, typing the name of the earlier defined routine (BELL – a previously defined simple program to ring the terminal bell) initiates a dictionary search to locate this routine's starting address which subsequentially is entered three times. The resulting SOUND routine contains machine code calls to the BELL routine which, itself, is composed of machine code. Of course, SOUND could also have been defined using a DO-LOOP, i.e.

```

: SOUND 3 1 DO BELL LOOP ;

```

where the numbers three and one set the upper and lower indices. If it were desirable to change the actual number of bell rings from some other program, this value could be defined as a VARIABLE – let's call it NOISE.

```
3 VARIABLE NOISE
```

In this case, the number three is first pushed on the stack, VARIABLE transfers the top number on the stack (the three) to a dictionary location named NOISE. If SOUND were now defined as :

```

: SOUND NOISE @ 1 DO BELL LOOP ;

```

the bell would again ring three times. In this case, when the word NOISE is encountered, its address is pushed on the stack, the @ is a simple program which goes to the address indicated on the top of the stack (that of NOISE) and replaces it with the actual value located at that address (the number three). At any future time, the value of a VARIABLE can be changed by "pushing" the new value onto the stack, followed by the address of the variable to be changed, generated by its name and an exclamation mark. To change NOISE to 5,

5 NOISE !

a number five is pushed onto the stack, NOISE pushes its address on the stack, and ! goes to the address indicated by the "top" number on the stack and deposits the next number. Now sound would ring the terminal bell five times.

A much less trivial program which could be written to scan and take data from a monochromator equipped with a DENCO SM2A stepper motor controller [2] (the SM2A takes a parallel number as an address, sends one of two stepper motors to this location and outputs an arrival flag when the address is reached) is given in Figure 6. Assume that the experimental system is configured so the SM2A is at I/O, port 5 and an analog to digital converter to acquire data is at I/O, port 7. Let us assume that, initially, a scan is designed from a starting stepper motor location of 2000 to a final location of 5000, taking data every 20 steps.

The program illustrated in figure 6 acts in the following manner. Line 1 defines a variable called START to be 2000, which is the starting location of the scan. The end of the scan is defined as 5000 in the next line. Line 3 defines the increment between data points. A variable called LOCATION where the next address is stored is defined in line 4. Colon, in line 5, puts the system in the compile mode, A/D7 will be the name of the module which when called will cause a 7 to be pushed onto the stack. INDEVICE will 'pop' it back off and use it as a device address to go and take a data point and push the data onto the stack. Line 6 defines a module INITIALIZE, START puts its address on the stack, @ replaces the address with the value at that address, LOCATION puts its address on the stack, '!' goes to the address specified by the number on the stack and deposits the second number and the net result value at START is put into LOCATION. Line 7 defines STEP to take values from LOCATION and INCREMENT adds them together and puts the result into LOCATION, i.e. LOCATION puts its address on the stack, @ replaces the top value on the stack with the number stored at the address, INCREMENT @ gets the value at INCREMENT and puts it in to the stack, and adds the top two stack numbers and pushes the result on the stack. LOCATION puts its address on the stack and ! goes to the address specified by the top number on the stack and deposits the second number. The module defined in line 8 takes a number from the stepper motor controller (assume device numbered 5) pushes on the stack, and pushes the value 10 on the stack and does a logical AND to see if the controllers flag is set, if this is true the A/D7 module will be called to input data, if the flag is not set, the program is returned to BEGIN–HERE. Therefore the DELAY module is a loop waiting for the stepper motor to arrive at its new location followed by a call to A/D7 data acquisition module. MOVE defined in line 9 gets the value stored at LOCATION pushes the device code onto the stack performs an outdevice (which uses the top stack number as a 1/0 port address to send the next value to call the STEP module (ie the value at LOCATION), this increments LOCATION by the INCREMENT value and finally calls DELAY and waits for a flag from the stepper motor controller to signal its arrival at the desired address and then takes a data point. The final line shown in the example called SCAN itself calls the INITIALIZE module (which sets LOCATION to the START location for the Scan) it also calls MOVE (which sends this value to the motor LOCATION controller, increments the value stored in LOCATION by INCREMENT and calls DELAY which waits for the motor to arrive and then takes a data point). Next the incremented value from LOCATION is placed on the stack (LOCATION) followed by the STOP

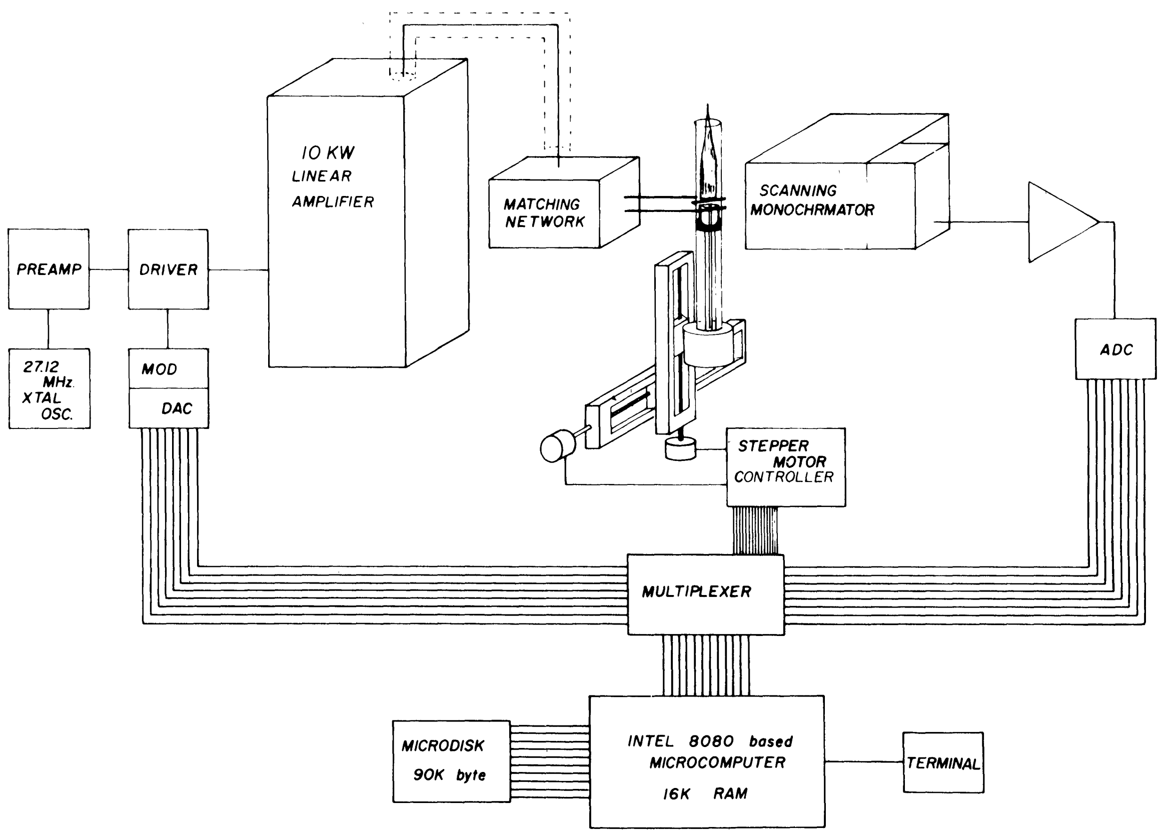

*Figure 8. A schematic of the inductively coupled plasma emission spectrometer in which the microcomputer is used to control radio frequency power and 'flame' positioning as well as to monitor light intensity.*

value (STOP @ ), the two are compared to > to see if the incremented value at LOCATION is larger then the STOP value, if it is the program ends, if not, it repeats the process starting at BEGIN–HERE.

It should be noted that whilst many variables have been pushed on the stack, only the data will remain, since each time a value is used it is 'popped' (removed) from the stack. If a different spectra region is to be scanned i.e. from 3000 to 6500 with 10 increments the variables need only be changed thus

```
3000 START !
6500 STOP  !
  10 INCREMENT !
```

and type SCAN, system will now scan from 3000 to 6500 taking data every 10 steps.

While the code might look a little strange at first, it quickly becomes very easy to work with. The SCAN program of Figure 6 could be combined with other modules as shown in Figure 5 to perform some more complex experimental function. Each module of the program can be easily tried out to ensure that it is operational before proceeding with the next.

Presently, CONVERS is being used in the authors' laboratories for a variety of spectrochemical investigations, including laser excited optoacoustic spectroscopy (Figure 7) and inductively coupled plasma optical emission spectroscopy (Figure 8). Rather complex interactive control and data acquisition programs have been easily implemented. Memory requirements and operating speed have been found to be far superior to conventional approaches. Additionally, new system users have encountered a difficulty in utilising previously developed custom software for a particular experiment even when documentation was vague.

### Discussion

The authors hope that this short introduction to only a few of the concepts employed in CONVERS will generate interest in its capabilities. A much more complete discussion is available in the form of a user's manual [3] available from the authors.

ACKNOWLEDGEMENTS
The development of the CONVERS system was partially supported by the Office of Naval Research and a Alfred P. Cloan Foundation Research Fellowship to M. Bonner Denton.

REFERENCES

[1] C. Moore, *Astronomical Astrophysics Supplemental,* 15,(1974) 497.
[2] M.B. Denton, J.D. Mack, M.W. Routh and D.B. Swartz, *American Laboratory,* 8,69 (1976).
[3] CONVERS An Interpretive Compiler, developed by Scott B. Tilden and M. Bonner Denton, Department of Chemistry, University of Arizona, Tucson, Arizona 85721, USA.

### The use of a microcomputer for flexible automation of a liquid chromatograph

**A.D. Mills, I. Mackenzie and R.J. Dolphin***

*Philips Research Laboratories, Redhill, Surrey, RH1 5HA, U.K.*

### Introduction

Microprocessors are being used to add inexpensive automatic control and data handling facilities to a variety of chemical instruments. With a microcomputer it is now possible to realise the flexibility formerly available only with a relatively large and expensive minicomputer in an instrument little different in size and cost from one controlled by inflexible hardware. In many ways chromatography is an ideal process for such automation. Most instruments are given a high workload and, although many applications may be routine and repetitive, the versatility of the technique requires an instrument which can easily be used in a variety of modes.

In addition to improving the convenience to the user, automation of a liquid chromatrograph should enhance the performance of the instrument. Some aspects of high performance liquid chromatography (HPLC) which can benefit in this way are as follows:

(1) Accurate control of solvent flow rate will compensate for changes in pressure drop and lead to more reliable retention times.
(2) The composition of the mobile phase can be accurately controlled in either isocratic or gradient elution chromatography using, for example, a proportioning valve on the low pressure side of the pump.
(3) Automatic sampling can be operated in a variety of modes to process a number of samples without supervision. It is also more precise than manual injection.
(4) A built-in data handling facility can present the analyst with an easily read post-run report of the analytical results with accurate peak area measurements even for peaks which are poorly resolved.

Although liquid chromatographs incorporating microprocessors for control and data handling purposes are commercially available, these instruments are, so far, relatively inflexible. This paper describes, in detail, the automation of a liquid chromatograph using an inexpensive general purpose microcomputer, which has previously been applied in atomic absorption spectrophotometry [1] and for column switching in HPLC [2].

Figure 1 illustrates the interconnection of the chromatograph and the microcomputer which controls the mobile phase flow rate, operates an automatic sampler and analyses data from the detector. The control and data handling functions are integrated in a program which enables the user to communicate with the instrument, in plain English, via a visual display unit (VDU) or teletypewriter keyboard. A variety of operational modes is offered, giving the analyst an opportunity to establish the best conditions for a particular separation before leaving the instrument to perform its given tasks without further interaction.

### The microcomputer

### Hardware

The computer is a general purpose instrument constructed using a set of ready made circuit cards (Philips, Science and

**Present address: Pye Unicam Ltd., York Street, Cambridge, CB1 2PX, U.K.*

